# Reliable Activation of Immature Neurons in the Adult Hippocampus

**DOI:** 10.1371/journal.pone.0005320

**Published:** 2009-04-28

**Authors:** Lucas A. Mongiat, M. Soledad Espósito, Gabriela Lombardi, Alejandro F. Schinder

**Affiliations:** Laboratory of Neuronal Plasticity, Leloir Institute – CONICET, Buenos Aires, Argentina; University of Washington, United States of America

## Abstract

Neurons born in the adult dentate gyrus develop, mature, and connect over a long interval that can last from six to eight weeks. It has been proposed that, during this period, developing neurons play a relevant role in hippocampal signal processing owing to their distinctive electrical properties. However, it has remained unknown whether immature neurons can be recruited into a network before synaptic and functional maturity have been achieved. To address this question, we used retroviral expression of green fluorescent protein to identify developing granule cells of the adult mouse hippocampus and investigate the balance of afferent excitation, intrinsic excitability, and firing behavior by patch clamp recordings in acute slices. We found that glutamatergic inputs onto young neurons are significantly weaker than those of mature cells, yet stimulation of cortical excitatory axons elicits a similar spiking probability in neurons at either developmental stage. Young neurons are highly efficient in transducing ionic currents into membrane depolarization due to their high input resistance, which decreases substantially in mature neurons as the inward rectifier potassium (Kir) conductance increases. Pharmacological blockade of Kir channels in mature neurons mimics the high excitability characteristic of young neurons. Conversely, Kir overexpression induces mature-like firing properties in young neurons. Therefore, the differences in excitatory drive of young and mature neurons are compensated by changes in membrane excitability that render an equalized firing activity. These observations demonstrate that the adult hippocampus continuously generates a population of highly excitable young neurons capable of information processing.

## Introduction

The dentate gyrus is the main gateway to the hippocampus and it constitutes a primary neurogenic niche of the adult brain. Neural progenitor cells of the subrgranular zone give rise to dentate granule cells (DGCs) that develop and mature over several weeks. A substantial fraction of those newly generated neurons become integrated in the hippocampal network and are then maintained throughout adulthood [1], [2]. In recent years, we and others have utilized *in-vivo* retroviral labeling or transgenic technologies to express fluorescent reporters in new DGCs of the adult mouse hippocampus to study their anatomical and functional maturation [3]–[6]. It was thus shown that developing DGCs follow a precise sequence to establish their afferent connectivity and functional maturation. Developing neurons are initially contacted by GABAergic terminals and later by glutamatergic axons. In parallel, their membrane resistance decreases and excitability becomes mature [4], [7]–[10]. When fully developed, adult-born neurons achieve a functional profile that is indistinguishable from that of all other DGCs as reflected by their inputs, intrinsic membrane properties and firing behavior [11], [12]. In addition, adult-born DGCs form functional glutamatergic synapses onto dentate gyrus interneurons and CA3 pyramidal cells [13], [14]. These observations indicate that new neurons receive, process and convey information onto target neurons, and might therefore participate in hippocampal function.

The participation of newly generated neurons in hippocampal processing has been highlighted by recent reports whereby reduced or blocked neurogenesis impair performance in hippocampus-dependent learning tasks [15]–[19]. Studies using the expression of immediate early genes as indicators of neuronal activity suggest that new DGCs are preferentially active during behaviors that involve spatial processing and memory formation [20]–[22]. Yet, the developmental stage at which new neurons become functionally significant and how those unique properties might impact in their function remains unknown. Young neurons exhibit high input resistance and increased susceptibility for the induction of long-term potentiation (LTP) of entorhinal glutamatergic inputs [23]–[25]. Based on these properties it has been proposed that they might play unique roles in hippocampal function [26]–[28].

To put forward the hypothesis that young neurons behave as a distinct neuronal population within the active hippocampal networks it is crucial to determine when they become engaged in firing activity, and how their activity compares to the remaining units of the network. We have used retroviral labeling and electrophysiological tools to study intrinsic membrane properties and input-output conversion in developing DGCs of the adult mouse hippocampus. We found that three- to four-week-old neurons display weak glutamatergic inputs, yet spike reliably in response to perforant path stimulation. In these immature neurons ionic currents are efficiently converted into membrane depolarization due to their high input resistance. As neurons mature the membrane resistance decreases, with a consequent reduction in excitability. In addition, we found a developmental increase in the inward rectifier K^+^ conductance (Kir) that highlights Kir channels as regulators of the excitability in newborn DGCs in the adult hippocampus.

## Results

The activity of the hippocampal network can only be modified by neurons that can integrate incoming signals to produce a firing behavior and alter the state of postsynaptic targets. Firing is ultimately shaped by the concerted action of synaptic integration and excitability. To investigate the impact of developing DGCs in the adult hippocampal network, a retrovirus encoding GFP driven by a strong promoter was used to label adult-born DGCs and acute brain slices were prepared 18 to 29 days after retroviral injection (dpi), at which time neurons are still immature, yet afferent excitatory connections become established [4], [9], [29]. An additional experimental group of GFP^+^ neurons that reached functional maturity was included for comparison (42 to 56 dpi). Intrinsic properties, excitatory inputs and spiking were characterized by electrophysiological recordings in GFP^+^ neurons and compared with those of unlabeled mature neurons of the outer granule cell layer, that are mostly generated during perinatal development (“mature” group; see Methods section).

### Young neurons are highly excitable

**Figure 1:**
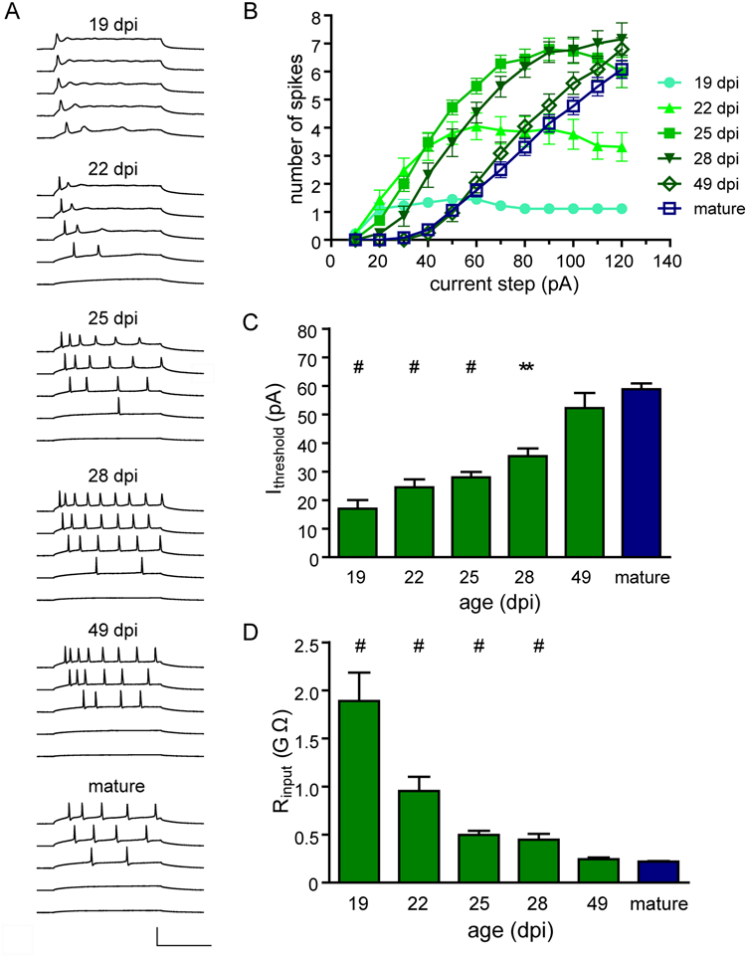
Young neurons are highly excitable. (A) Whole-cell current clamp recordings in neurons of different ages, as indicated on top of each panel. Spiking was elicited by depolarizing current steps of increasing amplitude (step = 10 pA). Each panel shows a subset of five representative traces with steps 10, 30, 50, 70 and 90 pA (from bottom to top). Scale bars: 100 mV, 100 ms. (B) Repetitive firing quantified as the number of spikes elicited by increasing current steps. Sample sizes are N = 10 (19 dpi), 20 (22 dpi), 29 (25 dpi), 13 (28 dpi), 24 (49 dpi) and 51 (mature). (C) Current threshold to elicit the first spike for the experiments shown in (B). (*#*) denotes *p*<0.001, and (**) denotes *p*<0.01 when compared to mature values. Statistical analysis was done by ANOVA followed by a Bonferroni's test. (D) Input resistance as a function of age. (*#*) denotes *p*<0.001 when compared to mature as analyzed by a Kruskal–Wallis test followed by a post hoc Dunn's test, with N = 13 (19 dpi), 23 (22 dpi), 23 (25 dpi), 18 (28 dpi), 18 (49 dpi) and 85 (mature).

The excitability of developing neurons in the adult dentate gyrus was investigated by monitoring passive and active membrane properties in whole-cell recordings. Injection of current steps of small amplitude readily generated action potentials in young DGCs, whereas increasingly larger currents were required to reach the membrane threshold for action potential in more mature neurons (Figure 1A–1C; Table 1). This is in agreement with previous observations that intrinsic excitability is higher in immature neurons [4], [7], [8], [23], [24], [30]. The dynamic properties of spikes also displayed a marked time-dependent maturation. Eighteen to 20 dpi neurons (“19 dpi” group) could only generate single action potentials with immature characteristics in response to prolonged membrane depolarizations, whereas older DGCs exhibited a progressive increase in the number of spikes. Interestingly, 24–29 dpi neurons fired with higher efficacy than younger or older DGCs (Figure 1B).

**Table 1:** Electrical properties of young and mature neurons

	young	mature	*p*
**INTRINSIC PROPERTIES**			
**R_in_ (MΩ)**	519±30 (68)	224±7 (89)	**<0.0001**
**C_m_ (pF)**	30.6±1.0 (68)	56.9±2.1 (89)	**<0.0001** e
**Tau (ms)** a	32.5±2.4 (25)	34.0±2.0 (25)	**0.634**
**V_resting_ (mV)**	−75.6±0.5 (42)	−80.6±0.5 (60)	**<0.0001**
**I_threshold_ (pA)**	29.0±1.7 (42)	58.8±2.5 (60)	**<0.0001** e
**V_threshold_ (mV)**	−42.8±0.9 (38)	−38.4±0.9 (55)	**0.0011**
**AHP (mV)**	−57.7±0.5 (23)	−59.8±0.4 (23)	**0.0024** e
**spike amplitude (mV)**	113.8±0.8 (23)	118.8±0.4 (23)	**<0.0001**
**spike slope (V/s)**	266±8 (23)	309±6 (23)	**0.0002**
**Max gNa^+^ (nS)** b	307±20 (26)	423±6 (29)	**<0.0001**
**Max gK^+^ (nS)** c	189±13 (26)	288±16 (29)	**<0.0001**
**gKir (nS)** d	1.73±0.32 (16)	5.18±0.34 (20)	**<0.0001**
**SYNAPTIC PROPERTIES**			
**EPSC Rise (ms)**	3.64±0.13 (40)	3.37±0.13 (40)	**0.16**
**EPSC Decay (ms)**	21.8±0.9 (40)	25.1±0.9 (40)	**0.009**
**EPSP Rise (ms)**	7.01±0.18 (42)	5.83±0.18 (39)	**<0.0001**
**EPSP Decay (ms)**	34.0±1.8 (43)	37.3±2.0 (42)	**0.22**

a Tau measured by fitting a single exponential equation to the membrane potential response to a current pulse (10 pA)

b gNa^+^ measured at V_h_ = −20 mV

c gK^+^ measured at steady state at V_h_ = 70 mV

d gKir^+^ measured as described in Methods

e Welch's correction for differences in variances was applied.

Mean±SEM are shown, with cell numbers in parentheses. Statistical analyses were performed by two-tailed t-tests.

The input resistance of a neuron (R_in_) reflects the cell size and the density of ion channels open at resting. High values of R_in_ are associated with an enhanced excitability, as small inward currents can elicit large membrane depolarizations. Young neurons displayed high values of R_in_ (in the GΩ range) that decreased as they reached mature developmental stages (Figure 1D). The age-dependent decline in R_in_ could, by itself, explain the larger current needed to elicit spiking in mature neurons. However, the increase in spiking frequency that accompanies neuronal maturation also suggests changes in spike properties, as addressed below.

### Weak glutamatergic inputs elicit spikes in young neurons

**Figure 2:**
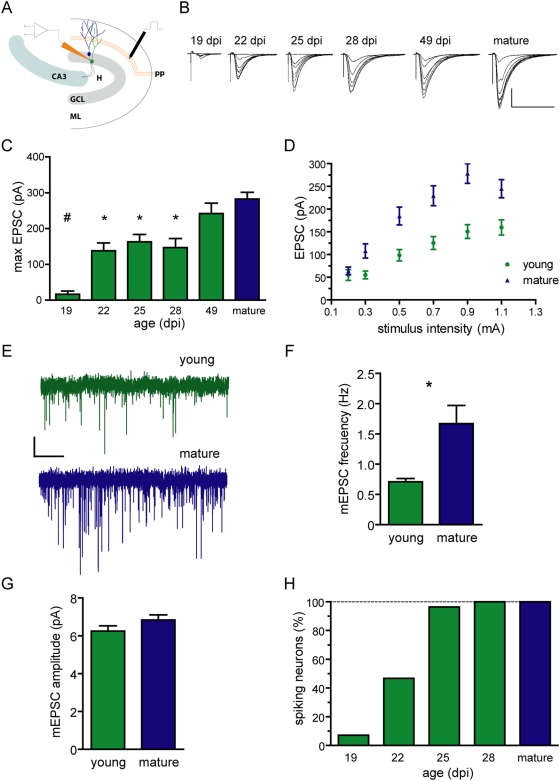
Spikes in young neurons elicited by weak glutamatergic inputs. (A) Illustration depicting electrophysiological recordings of postsynaptic responses in acute hippocampal slices. Stimulation was performed in the medial perforant path (pp), and whole-cell recordings were obtained in young (green) or mature (blue) granule cells. GCL, granule cell layer; ML, molecular layer; H, hilus. (B) Example traces of EPSCs evoked by increasing stimulus strength (0.2–1 mA, 50 µs) obtained from DGCs of different developmental stages (shown on the top). Each trace is an average of five epochs. Scale bars: 100 pA, 50 ms. (C) Maximal peak EPSC amplitude for young and mature DGCs. (#) denotes *p*<0.001, and (*) denotes *p*<0.05 when compared to mature, as analyzed by ANOVA followed by a Bonferroni's post-hoc comparison, with N = 9 (19 dpi), 9 (22 dpi), 14 (25 dpi), 10 (28 dpi), 10 (49 dpi) and 41 (mature). (D) Peak EPSC amplitude vs. stimulus intensity in young (21–29 dpi) and mature DGCs. Two-way ANOVA revealed a significant effect of both neuronal age and stimulus intensity (*p*<0.0001 for both), with N = 27 for both groups. (E) Representative traces of miniature postsynaptic currents obtained in presence of 0.5 µM tetrodotoxin. Scale bars: 2.5 pA, 5 s. (F, G) Frequency and amplitude of mEPSCs. (*) denotes *p* = 0.016; for amplitude *p* = 0.14; N = 8 for both. (H) Fraction of spiking neurons as a function of age. Spiking was measured in whole-cell current clamp and was elicited by stimuli that rendered maximal EPSC amplitudes, with N = 14 (19 dpi), 15 (22 dpi), 28 (25 dpi), 21 (28 dpi) and 59 (mature).

To determine whether an excitatory drive can trigger action potentials in immature DGCs, postsynaptic responses to afferent glutamatergic stimulation were recorded in the presence of GABA receptor antagonists (picrotoxin 100 µM and CGP 55845 100 nM). Excitatory postsynaptic currents (EPSCs) were evoked by increasing stimulus intensities delivered to the medial perforant path (Figure 2A). Peak EPSC amplitudes were very weak throughout the stimulus range in 19 dpi neurons but increased substantially with age, reaching a maximal amplitude by 49 dpi (Figure 2B and 2C). Therefore, all functional studies in developing neurons were carried out in DGCs aging 21 to 29 dpi, hereafter the “young” group. The small amplitudes of evoked EPSCs recorded in young neurons (Figure 2D) might be due to presynaptic mechanisms such as a small number of synaptic contacts and/or low release probability, or to postsynaptic mechanisms such as reduced amplitude of unitary postsynaptic currents [31]. Recordings of miniature excitatory postsynaptic currents (mEPSCs) revealed a reduced frequency in young cells with no differences in amplitude, indicating presynaptic rather than postsynaptic differences (Figure 2E–2G). Together with the morphological evidence of low spine density in immature DGCs (21 dpi: 0.78±0.10 spines/µm, mature: 2.40±0.06 spines/µm, N = 19 neurons for both; V.C. Piatti and A.F.S. unpublished observations; see also [29]) our observations indicate that the weak glutamatergic input is due to a limited number of afferent terminals impinging onto young neurons.

Stimuli that evoked the larger EPSC amplitudes were selected to assess spiking by an excitatory drive. DGCs were practically unable to spike by 19 dpi most likely due to their weak excitatory input combined with their limited ability to generate action potentials (Figure 1B and Figure 2H). As afferent excitation strengthened (>21 dpi), the proportion of young DGCs exhibiting spikes in response to robust stimuli increased substantially. These observations indicate that, in principle, immature neurons of the adult hippocampus might be recruited by cortical afferent activity.

### Young DGCs fire with high efficacy

**Figure 3:**
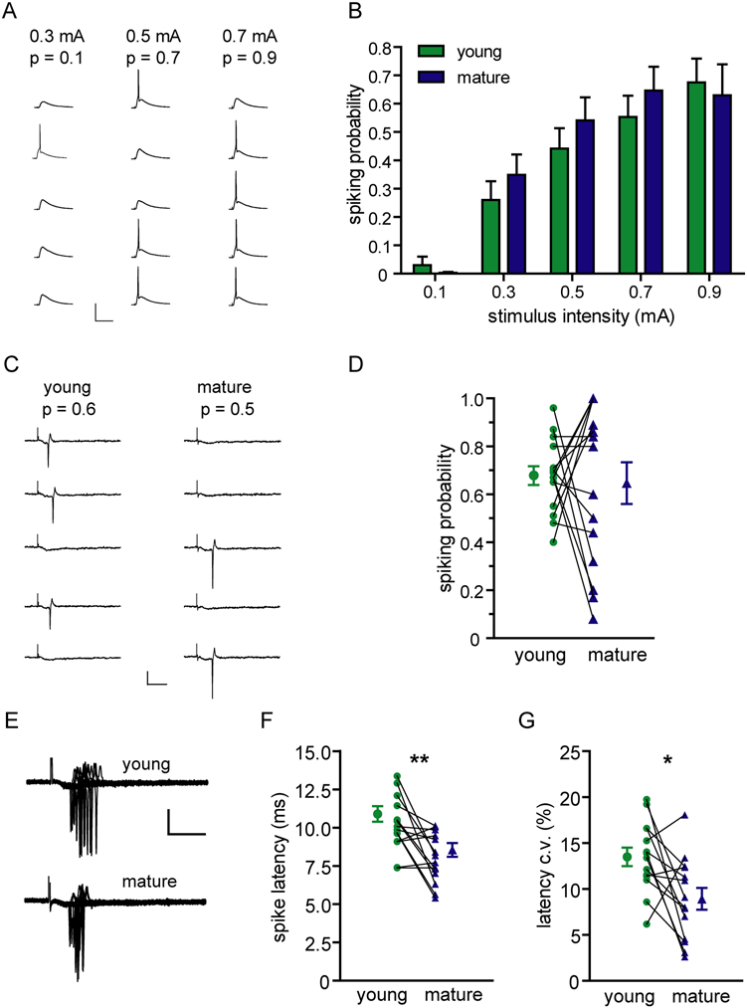
Equalized firing in young and mature neurons. (A) Example of a recording performed to measure spiking probability in response to presynaptic stimuli of different intensities in the same neuron. Each column shows typical membrane potential traces in response to stimuli repeated at low frequency (0.07 Hz). Probabilities are shown on the top. Scale bars: 50 mV, 100 ms. (B) Quantitative analysis of spiking probability. Each experiment involved consecutive recordings of a young and a mature neuron at all stimulus intensities in the same slice. Two-way ANOVA revealed no differences between young and mature cells (*p* = 0.36), but a significant effect was observed for stimulus intensity (*p*<0.0001). Bars represent mean±SEM of 39 neuron pairs from 27 slices. (C) Example of paired measurements of spiking probability in a young and a mature neuron in dual cell-attached recordings. Action currents elicited by repetitive stimuli delivered at low frequency (15 repetitions at 0.06 Hz) were recorded to simultaneously assess spiking probability in both neurons. A subset of 5 epochs is shown. Scale bars: 20 pA, 10 ms. (D) Paired analysis of spiking probability measured in 15 experiments. Each recorded pair is connected by a line; mean±SEM are shown. A paired two-tailed *t*-test revealed no statistical differences between the groups (*p* = 0.77). (E) Overlay of action currents in simultaneous cell-attached recordings of young and mature neurons. Note the enhanced jitter in young DGCs. Scale bars: 20 pA, 10 ms. (F) Measurements of spike latency analyzed by a paired two-tailed *t*-test; (**) denotes *p* = 0.0062 with N = 14 pairs. (G) Spike jitter measured as the relative coefficient of variation (c.v.) for individual action currents; (*) denotes *p* = 0.014 with N = 14 pairs.

Since young and mature DGCs display distinct excitability, spike properties and input strength, it would be predictable that they are differentially recruited into an active network. To address this question spiking probability of young (21–29 dpi) and mature DGCs were measured in current clamp, while repetitive stimuli of increasing strength were delivered at low frequency to the medial perforant path (Figure 3A). Surprisingly, young and mature neurons exhibited a similar spiking probability at all stimulus strengths (Figure 3B). A more precise determination of the firing behavior was obtained by dual cell-attached recordings of young and mature neurons, where the firing probability was measured in pairs of neurons stimulated under the same conditions. This approach brings the advantages that: 1) the intracellular milieu remains unaltered by the recoding pipette; 2) the resting potential of the cells, which greatly influences firing behavior, remains at physiological values; 3) all data are collected in a paired fashion and systematic errors are minimized. In each experiment, action currents elicited by repetitive stimuli delivered at low frequency were recorded to simultaneously assess spiking probability in both neurons. Consistent with the results presented above, no differences in overall firing behavior were observed (Figure 3C and 3D). Yet, differences were found in the stimulus-to-spike delay; young neurons displayed longer spike latency (Figure 3E–3F), consistent with the slower EPSP rise time (Table 1). In addition, spike timing precision increased with neuronal maturation (Figure 3G), consistent with previous observations in the developing brain [32].

**Figure 4:**
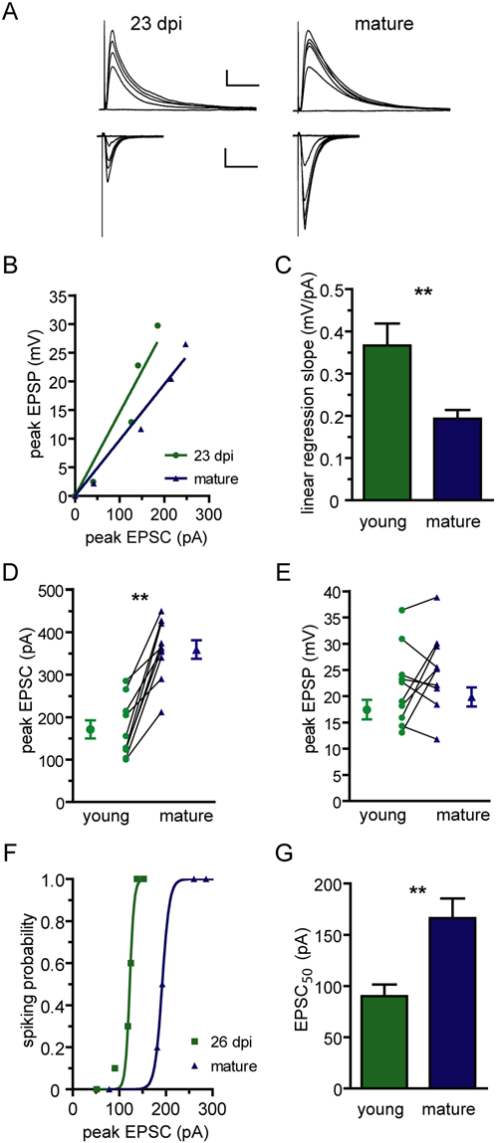
Efficient input-output conversion in young neurons. (A) Typical traces recorded in a young and a mature neuron displaying evoked EPSPs (upper panels) and EPSCs (lower panels) for the same series of increasing stimuli (0.1–1 mA) delivered to the medial perforant path. Scale bars: 5 mV/50 pA, 50 ms. (B) Data from a representative experiment showing EPSP / EPSC pairs and their linear regression curves. (C) Average slope of linear regressions. Statistical difference was analyzed by a two-tailed *t*-test with Welch's correction for different variance; (**) denotes *p* = 0.0031, N = 40 (both). (D) Simultaneous voltage clamp recordings displaying pairs of EPSC amplitudes evoked by the same presynaptic stimuli. A paired *t*-test revealed significant differences; (**) denotes *p* = 0.002, with N = 10 cell pairs. (E) Simultaneous current clamp recordings carried out in the same cells shown in (D). No significant differences were observed in peak EPSP amplitude between young and mature neurons (*p* = 0.22, N = 10). (F) Representative plot of spiking probability vs. EPSC amplitude for a 26 dpi and a mature DGC. Lines correspond to sigmoid fittings y = exp [m.(x−x_50_)] / {1+exp [m.(x−x_50_)]}, with m: slope, x: EPSC, y: p spiking and x_50_: EPSC for p = 0.5. (G) Mean of interpolated EPSC values required to evoke a spiking probability of 0.5 (EPSC_50_). Statistical difference was analyzed by a two-tailed *t*-test with Welch's correction; (**) denotes *p* = 0.0017, N = 23 (both).

It is surprising that young and mature DGCs have different functional properties but display similar firing behavior. To better understand this phenomenon we investigated how synaptic currents are transduced into membrane depolarization. Current- and voltage clamp recordings were combined to monitor subthreshold postsynaptic responses evoked by afferent stimulation with increasing strength. Thus, each stimulus generated an EPSP / EPSC pair whereby a given postsynaptic depolarization was associated to a particular synaptic current (Figure 4A and 4B). Young neurons required about half the synaptic strength as mature DGCs to depolarize the membrane to a similar extent (Figure 4C). This observation was corroborated in simultaneous double patch recordings, where the same stimulus elicited similar EPSPs but smaller EPSCs in young DGCs (Figure 4D and 4E). We also examined whether the high efficacy of membrane depolarization was effective during suprathreshold depolarizations in young DGCs. Thus, EPSC amplitude and the corresponding spiking probability were monitored for each stimulus strength. Similarly to the subthreshold situation, young neurons demanded about half the synaptic strength to reach a similar spiking probability when compared to mature DGCs (Figure 4F and 4G, Figure S1).

### Late onset of inward rectifier K^+^ conductance in developing DGCs

**Figure 5:**
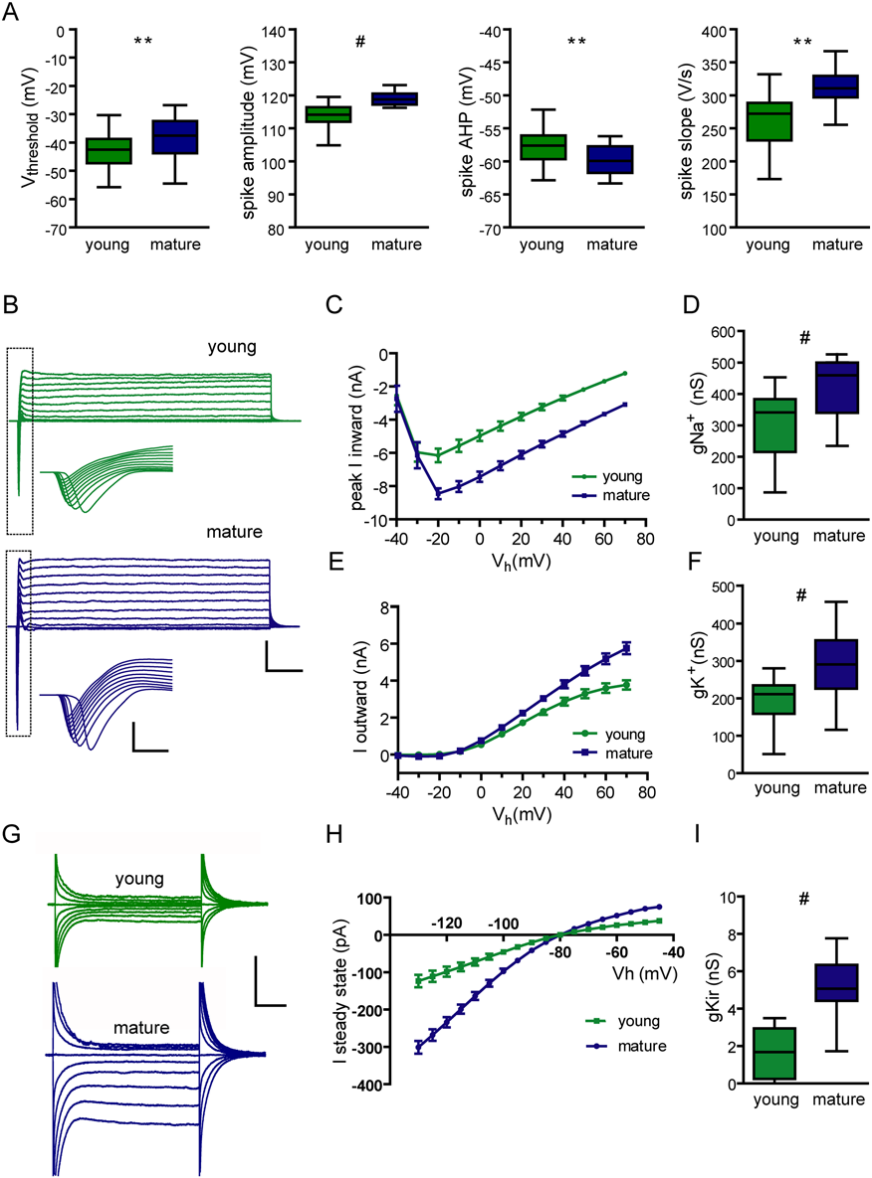
Active membrane properties of young neurons. (A) Characterization of action potentials recorded in young and mature neurons. Spiking threshold, N = 38 (young) and 55 (mature); (*) denotes *p* = 0.011. Spike amplitude, N = 23 (both), (#) denotes *p*<0.0001. Spike slope measured from the 10–90% rising phase, N = 23 (both), (#) denotes *p* = 0.0002. AHP measured as the absolute peak value, N = 23 (both), (**) denotes *p* = 0.0024. Box plots depict the median (line), 25–75% percentile (box limits) and the maximum and minimum values (whiskers). Statistical analysis was done using a two-tailed *t*-test. (B) Typical examples of inward (downward deflections) and outward (upward deflections) currents recorded in response to depolarizing voltage steps (−40 to 70 mV, step 10 mV, 200 ms) in a 25 dpi (green) and a mature DGC (blue). Scale bars: 5 nA, 50 ms. Insets depict expanded views of the squared areas (scales: 5 nA, 1 ms). (C) I–V curve for voltage-gated Na^+^ currents, displaying different amplitudes for young and mature cells (ANOVA, *p*<0.0001, N = 26 young and 29 mature DGCs). (D) Na^+^ conductance measured at V_h_ = −20 mV; (#) denote *p*<0.0001. (E) I–V curve for voltage-gated K^+^ currents measured at steady state showing significant differences (ANOVA, *p*<0.0001). (F) K^+^ conductance measured at V_h_ = 70 mV; (#) correspond to *p*<0.0001. (G) Inward rectifying currents evoked by current steps from −40 to −130 mV (step 5 mV, 100 ms) recorded from a 23 dpi and a mature DGC. Scale bars: 200 pA, 20 ms. (H) Average I–V plots displaying inward rectifier K^+^ currents are significantly larger in mature neurons (*p*<0.0001), ANOVA with N = 19 (young) and 17 (mature). (I) K^+^ inward-rectifier conductance; (#) denotes *p*<0.0001.

To better understand the observed differences in firing behavior we characterized action potentials, voltage-gated Na^+^ and K^+^ currents, and inward rectifier K^+^ currents (Kir), which contribute to neuronal excitability in subthreshold conditions [33], [34]. The membrane threshold for spiking was slightly hyperpolarized in young DGCs while spike amplitude, rise slope, and afterhyperpolarization (AHP) were more prominent in older cells (Figure 5A, Figure S2, Table 1). The amplitude of voltage-activated currents also increased according to the maturation stage, yet a slower pace was observed in the development of Kir currents when comparing young vs mature neurons. Kir conductance increased by ∼300% whereas voltage-gated Na^+^ and K^+^ currents reached near plateau levels in young neurons, increasing by only ∼50% (Figure 5B–5I, Figure S3). These observations suggest an ongoing homeostatic regulation that maintains a high degree of excitability in immature DGCs by boosting currents involved in spike generation while limiting the development of Kir currents.

**Figure 6:**
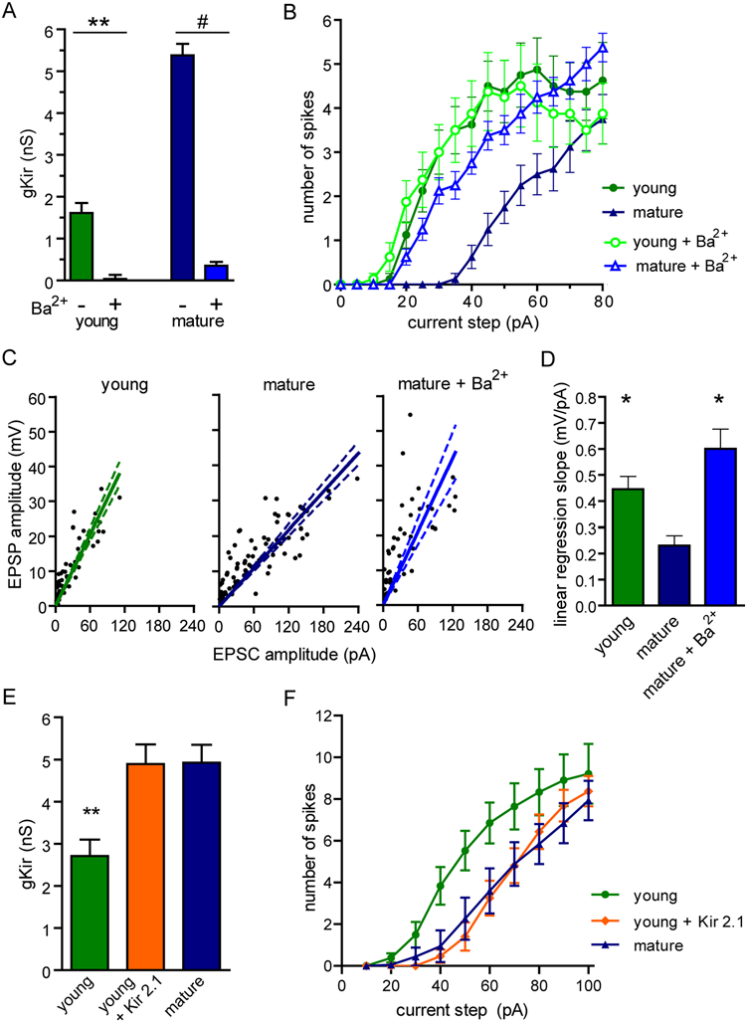
Control of the excitability of newborn cells by Kir channels. (A) Kir conductance in the absence or presence of BaCl_2_. (**) denote *p*<0.001, (#) denote *p*<0.0001 as analyzed by ANOVA followed by a Bonferroni's test, with N = 24 (young), 8 (young+Ba^2+^), 28 (mature) and 23 (mature+Ba^2+^). (B) The number of spikes elicited by depolarizing current steps (5 pA, 200 ms) reveals that Kir blockade by Ba^2+^ enhances suprathreshold excitability of mature but not young neurons. Statistical differences were found between young vs. mature (*p*<0.01), mature vs. mature+Ba^2+^ (*p*<0.05) and mature vs. young+Ba^2+^ (*p*<0.01), but not young vs. young+Ba^2+^ (p = 0.11). N = 8 (for all groups), analysis performed by ANOVA for repeated measures followed by a Bonferroni's test. (C) Kir blockade by Ba^2+^ increases subthreshold excitability assessed as EPSP / EPSC ratios. N = 16 (young) and N = 14 (mature and mature+Ba^2+^). Linear regression plots and the 95% confidence interval are shown (dotted lines). (D) Average slope of linear regressions. (*) denotes *p*<0.05 when compared to mature DGCs (ANOVA followed by Bonferroni's test). (E, F) Overexpression of Kir 2.1 in young DGCs (28–29 dpi). (E) Significant increase in Kir conductance by Kir 2.1 overexpression. (**) denote *p*<0.001 and (#) denote *p*<0.0001 as analyzed by ANOVA followed by a Bonferroni's test, with N = 13 (young–controls –), 12 (young+Kir 2.1) and 9 (mature). (F) The number of spikes elicited by depolarizing current steps (10 pA, 400 ms) indicates that Kir overexpression reduces suprathreshold excitability in young DGCs. Statistical differences were found between *young vs. mature* and *young vs. young+Kir 2.1* (p<0.01 for both). Analysis performed by ANOVA for repeated measures followed by a Bonferroni's test with N = 11 (young), 9 (young+Kir 2.1) and 6 (mature).

The observations described above suggest that the activity of DGCs might be regulated by the level of Kir expression. To address this question neuronal excitability was studied in the presence of extracellular Ba^2+^ (200 µM), a well known blocker of Kir channels [34]–[36]. Application of Ba^2+^ effectively abolished the inward rectification current component in young and mature neurons (Figure 6A, Figure S4). To evaluate Kir contribution to the firing behavior, depolarizing currents were delivered to young and mature neurons in the presence and absence of Ba^2+^. As shown above (Figure 1B), young neurons displayed enhanced repetitive spiking for small injected current pulses. Ba^2+^ induced a significant leftward shift in the spiking curve of mature neurons without altering the responsiveness of young cells (Figure 6B). We then assessed how acute Kir blockade affects synaptic integration. Analysis of subthreshold depolarization in response to evoked synaptic currents revealed that Ba^2+^ produced an increase in the EPSP/EPSC slope of mature DGCs toward values that are similar to those of young neurons (Figure 6C and 6D). Therefore, Kir blockade elicits a young neuronal behavior in fully mature DGCs, equalizing their degree of excitability. To investigate the effects of accelerated Kir expression on the excitability of young neurons, Kir 2.1 was overexpressed by retroviral delivery into dividing progenitor cells [37]. Kir overexpression increased inward rectifying currents and decreased suprathreshold excitability, inducing mature-like properties in young neurons (Figure 6E and 6F). These findings strongly suggest that young neurons of the adult dentate gyrus can spike with high efficacy due to the delayed onset of Kir channels.

## Discussion

DGCs generated in the adult hippocampus receive functional afferents, spike in response to an excitatory drive, and release glutamate onto postsynaptic target cells (this work,[3], [4], [9], [11], [13], [14]). This complete set of functional properties endows newly generated neurons with the capacity to play a significant role in hippocampal function. In agreement with this view, exploratory behavior and spatial learning paradigms that involve various hippocampal areas displayed preferential recruitment of new neurons within the dentate gyrus [20], [21]. In the present work we have used a retroviral labeling technique that allows reliable birth dating of newly generated neurons. Although fully mature neuronal properties were not observed until 49 dpi, DGCs that have developed for three to four weeks received weak excitatory inputs yet were capable of spiking in response to an afferent excitation. This is due to the fact that young neurons are very efficient in transducing ionic currents into membrane depolarization. Together with the recent observation that new neurons establish synapses onto target cells before reaching maturation [13], [14], the data presented here supports the notion that young DGCs participate in information processing [21], [27], [28]. Whether or not they are preferentially recruited during hippocampus-dependent behaviors may depend on the integration of both excitatory and inhibitory inputs, an issue that remains to be investigated.

Kir channels are involved in the resting membrane potential and conductance, exerting a role in the regulation of cellular excitability [36]. They are conductive at the resting potential and, therefore, decrease the membrane resistance with a consequent reduction in excitability. Intrinsic excitability refers to the propensity of a neuron to fire action potentials when exposed to an input signal, and it is directly attributable to the suite of ion channels inserted into the plasma membrane [38], [39]. Young DGCs display a high input resistance, low threshold potential for spiking, low threshold currents, and large EPSPs relative to the weak glutamatergic input. The expression of Kir in DGCs has been previously observed by in situ hybridization [40], and it is now demonstrated in young and mature neurons by I–V curves and pharmacological blockade. Kir currents strongly influenced the resting membrane conductance, since application of Ba^2+^ increased membrane resistance of mature neurons close to young DGC values and enhanced their excitability.

Voltage-gated Na^+^ and K^+^ channels displayed a slight age-dependent increase, consistent with the observed changes in action potential threshold and shape. This minor increment indicates that voltage-gated currents were close to plateau levels in young neurons. Consistent with this notion we observed only a minor increase in the rising slope of the action potential, a parameter that reflects the density of Na^+^ channels [41]. Remarkably, Kir conductance was weak in young neurons and increased substantially in mature DGCs. The early expression of voltage-gated Na^+^ and K^+^ channels together with the delayed expression of Kir optimize excitability of young neurons. This mechanism might obey to a homeostatic requirement to maintain a firing pattern in neurons with immature glutamatergic inputs. Supporting this idea, a stimulus delivered to the medial perforant path elicited similar spiking probabilities in young and mature neurons. The involvement of homeostatic mechanisms in the control of intrinsic excitability has been investigated in detail [38], [39]. In developing neurons, the strength of voltage-gated currents can be tailored by the synaptic drive to stabilize the dynamic range of neuronal output [42], [43]. In addition, reduced inhibition was shown to be compensated by an increase in endogenous leak conductance thus maintaining firing activity unaltered [44]. Our results suggest a homeostatic compensation of intrinsic excitability mediated by Kir that equalizes firing properties of all active DGCs in the adult hippocampus. This mechanism may facilitate activity dependent competition for survival [45] or, alternatively, it may strengthen the integration of young neurons in the network at a time in which afferent excitatory synapses display enhanced plasticity [25].

## Materials and Methods

### Viral vectors

A replication-deficient retroviral vector based on the Moloney murine leukemia virus was used to express enhanced GFP (or Kir-IRES-GFP in experiments shown in Figure 6E and 6F) driven by a CAG promoter [11], [29]. Mouse Kir 2.1 cDNA was kindly provided by G. Lanuza. Retroviral particles were assembled using three separate plasmids containing the capside (CMV-vsvg), viral proteins (CMV-gag/pol), and transgene (CAG-GFP). Plasmids were transfected into 293T cells using Lipofectamine 2000 (Invitrogen, Carlsbad, CA). Virus-containing supernatant was harvested 48 h after transfection and concentrated by two rounds of ultracentrifugation.

### Subjects and stereotaxic surgery

Female C57Bl/6J mice, 6–7 weeks of age, were anesthetized (100 µg ketamine/10 µg xylazine in 10 µl saline/g). CAG-GFP expressing retrovirus was infused (0.9 µl in 7 min) into the dorsal area of the right dentate gyrus (coordinates from bregma: antero-posterior = −2 mm, lateral = 1.5 mm, ventral = 1.9 mm) using a microcapillary calibrated pipette (Drummond Scientific, Broomall, PA) as described previously [4]. Housing, treatments, surgery and euthanasia were carried out under conditions that fully comply with the National Institutes of Health (NIH, USA) guidelines.

### Electrophysiology

Experiments were carried out in 352 neurons from 92 mice. Mice were anesthetized and decapitated at 18 to 56 days post injection (dpi), as described below. Brains were removed into a chilled solution containing (in mM): 110 choline-Cl^−^, 2.5 KCl, 2.0 NaH_2_PO_4_, 25.0 NaHCO_3_, 0.5 CaCl_2_, 7 MgCl_2_, 20 dextrose, 1.3 Na^+^-ascorbate, 0.6 Na^+^-pyruvate, and 4.0 kynurenic acid. Horizontal slices (400-µm thick) were cut in a vibratome (Leica VT1200 S, Nussloch, Germany) and transferred to a chamber containing (in mM): 125.0 NaCl, 2.5 KCl, 2.0 NaH_2_PO_4_, 25.0 NaHCO_3_, 2 CaCl_2_, 1.3 MgCl_2_, 1.3 Na^+^-ascorbate, 3.1 Na^+^-pyruvate, and 10 dextrose (315 mOsm). Slices were bubbled with 95% O_2_/5% CO_2_ and maintained at 30°C for at least 1 hour before experiments started. Adjacent sections to the injection site were discarded to avoid effects of inflammation. Recordings were performed at 23 ± 2°C using microelectrodes (4–6 MΩ) pulled from borosilicate glass (KG-33; King Glass, Claremont, CA) and filled with (in mM): 120 K-gluconate, 20 KCl, 5 NaCl, 4 MgCl_2_, 0.1 EGTA, 10.0 HEPES, 4.0 Tris-ATP, 0.3 Tris-GTP, 10 phosphocreatine, Alexa Fluor 594 (5 µg/ml; Invitrogen), pH 7.3, and 290 mOsm. Recordings were obtained using an Axopatch 200B amplifier (Molecular Devices, Sunnyvale, CA), digitized (Digidata 1322A; Molecular Devices), and acquired at 20 KHz onto a personal computer using the p-Clamp 9 software (Axon CNS, Molecular Devices).

All experiments were carried out in neurons that showed action potentials in response to current injection through the patch pipette. Developing neurons expressing GFP were binned in the following age groups: 18–20 dpi (“19 dpi” group), 21–23 dpi (“22 dpi”), 24–26 dpi (“25 dpi”), 27–29 dpi (“28 dpi”), and 42–56 dpi (“49 dpi”). GFP^+^ cells of different ages were identified in the granule cell layer using FITC fluorescence optics (DMLFS; Leica). In previous works we have compared mature neurons born in 15-day-old embryos, 7-day-old pups and adult mice and found no significant functional differences [11], [12]. Therefore, GFP- neurons localized in the outer third of the granule cell layer were selected as mature controls [7], [24].

Whole-cell voltage-clamp recordings were performed at a holding potential (V_h_) of −70 mV, unless otherwise noted. Criteria to include cells in the analysis were co-labeling with Alexa Fluor 594 or visual confirmation of GFP in the pipette tip and absolute leak current <100 pA at V_h_. Series resistance was typically 10–20 MΩ, and experiments were discarded if higher than 25 MΩ. Membrane capacitance and input resistance were obtained from current traces evoked by a hyperpolarizing step of 10 mV. In current-clamp recordings the resting membrane potential was kept at −70 mV by passing a holding current. The threshold current for spiking was assessed by successive depolarizing current steps (5 or 10 pA; 500 ms) to drive the membrane potential (V_m_) from resting to 0 mV. Action potential threshold was defined as the point at which the derivative of the membrane potential dV/dt deviated from the mean baseline value by >2 standard deviations [46], and was calculated using routines developed in our laboratory (Figure S2). Voltage-dependent Na^+^ and K^+^ currents were measured after leak subtraction using a p/−6 protocol and automatic detection of the fast inward peak and the late outward plateau. Kir currents were measured as the steady state inward deflection in response to hyperpolarizing voltage steps (from −130 to −90 mV). Kir conductance was calculated after subtracting the leak conductance measured between −60 and −40 mV, where Kir channels are closed. Kir conductance calculated in this manner did not differ significantly from the one measured as the fraction of inward conductance with sensitivity to blockade by 200 µM Ba^2+^.

Extracellular stimulation of the medial perforant path was performed using concentric bipolar electrodes (50 µm diameter; Frederick Haer Company, Bowdoinham, ME) and an Iso-Flex stimulator (A.M.P.I.; Jerusalem, Israel). The stimulation electrode was placed orthodromically on the middle third of the molecular layer at >250 µm from the recorded cell. Functional inputs were assessed at stimulus strengths of 0.1–1.3 mA (50 µs) repeated at 15-s intervals. Excitatory postsynaptic currents (EPSCs) and potentials (EPSPs) were recorded in the presence of picrotoxin (100 µM) and CGP 55845 (100 nM), antagonists of GABA_A_ and GABA_B_ receptors. Peak amplitudes were calculated from the average of 5 traces (EPSCs) and 10–20 traces (EPSPs). Simultaneous cell-attached recordings were carried out under voltage clamp at 0 mV using pipettes with a high tip resistance (10–14 MΩ). Stimulus intensity was gradually increased (0.3–2 mA, 50 µs) and, for each cell, spiking probability was obtained after 10–20 stimuli. For each pair of cells, the lower stimulus intensity at which both (young and mature) neurons fired was selected for statistical analysis of spiking probability. Whole-cell recordings were carried out at the end of all experiments to verify granule cell phenotype by spiking properties and morphology after filling with a fluorescent dye. Only experiments in which spiking was detected in both neurons were considered for analysis. Recordings were discarded if the seal resistance reached values below 8 GΩ. The integrity of the cell-attached patch was further confirmed by the absence of fluorescent dye in the cytoplasm.

## Supporting Information

Figure S1 Spiking probability vs. input strength. Spiking probability vs. EPSC amplitude measured in young and mature DGCs. Input strength was binned into three categories according to the EPSC amplitude. (*) and (**) denote *p*<0.05 and *p*<0.01 by two-way ANOVA revealing a significant effect by age, with N = 23 (both).
(0.83 MB TIF)

Figure S2 Graphic analysis used to determine spiking threshold. (A) Representative action potentials from young (green) and mature neurons (blue). Scale bars: 50 mV, 5 ms. (B) The plot depicts the derivative of the membrane potential (dV/dt) in relation to the membrane potential (Vm). Arrows indicate spiking thresholds for a young and a mature DGCs.
(1.10 MB TIF)

Figure S3 Ontogeny of Kir currents in adult-born DGCs. (A) Average I–V curves, with N = 13 (8 dpi), N = 14 (17 dpi), N = 18 (24 dpi), N = 12 (29 dpi) and N = 15 (35 dpi). (B) Kir conductance calculated for the experiments shown in (A).
(1.10 MB TIF)

Figure S4 Kir blockade by extracellular Ba^2+^. (A) Example current traces of young (25 dpi) and mature neurons recorded in the absence (left) or presence (right) of BaCl_2_ (200 µM). Voltage steps from −45 to −130 mV (step 5 mV, 100 ms) for a 25 dpi and a mature neuron. Scale bars: 200 pA, 30 ms. (B) Mean I–V plots obtained from N = 24 (young), 8 (young+Ba^2+^), 28 (mature) and 23 (mature+Ba^2+^).
(0.96 MB TIF)
