# Supplementary figures and images for: Reliable Activation of Immature Neurons in the Adult Hippocampus
Source: PLoS One. 2009 Apr 28;4(4):e5320. doi: 10.1371/journal.pone.0005320 (PMC2670498; doi:10.1371/journal.pone.0005320)

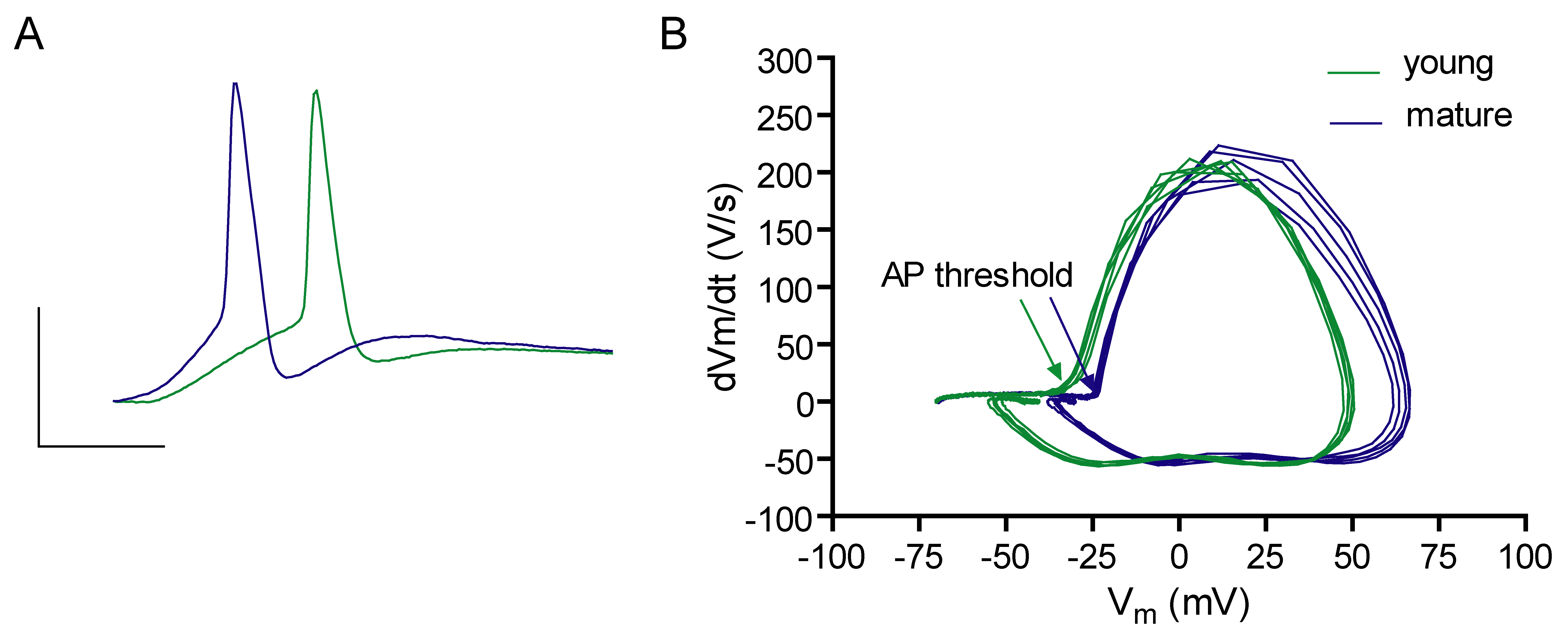

Supplement: Figure S1 — Spiking probability vs. input strength. Spiking probability vs. EPSC amplitude measured in young and mature DGCs. Input strength was binned into three categories according to the EPSC amplitude. (*) and (**) denote p<0.05 and p<0.01 by two-way ANOVA revealing a significant effect by age, with N = 23 (both). (0.83 MB TIF) [file pone.0005320.s001.tif]

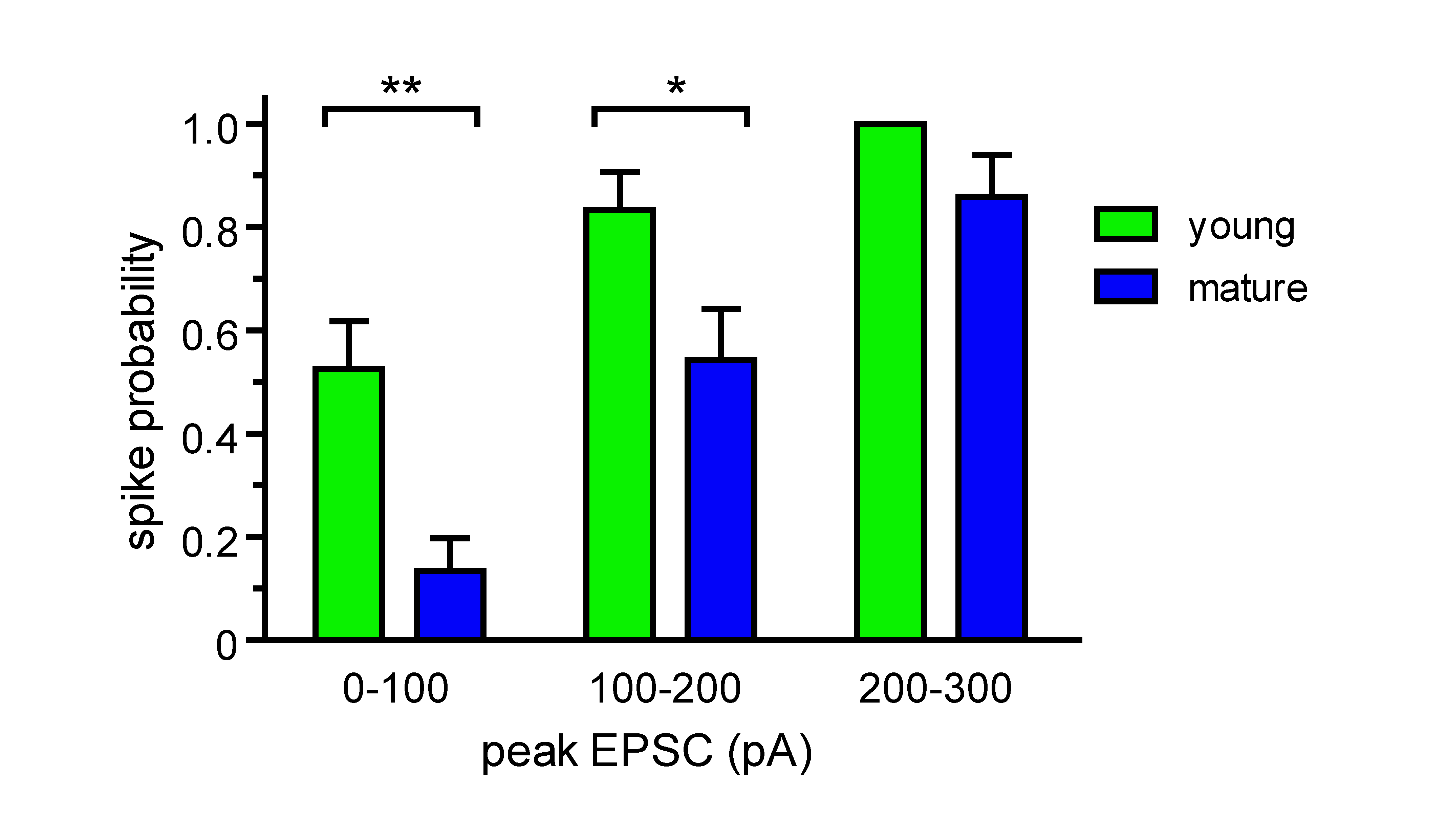

Supplement: Figure S2 — Graphic analysis used to determine spiking threshold. (A) Representative action potentials from young (green) and mature neurons (blue). Scale bars: 50 mV, 5 ms. (B) The plot depicts the derivative of the membrane potential (dV/dt) in relation to the membrane potential (Vm). Arrows indicate spiking thresholds for a young and a mature DGCs. (1.10 MB TIF) [file pone.0005320.s002.tif]

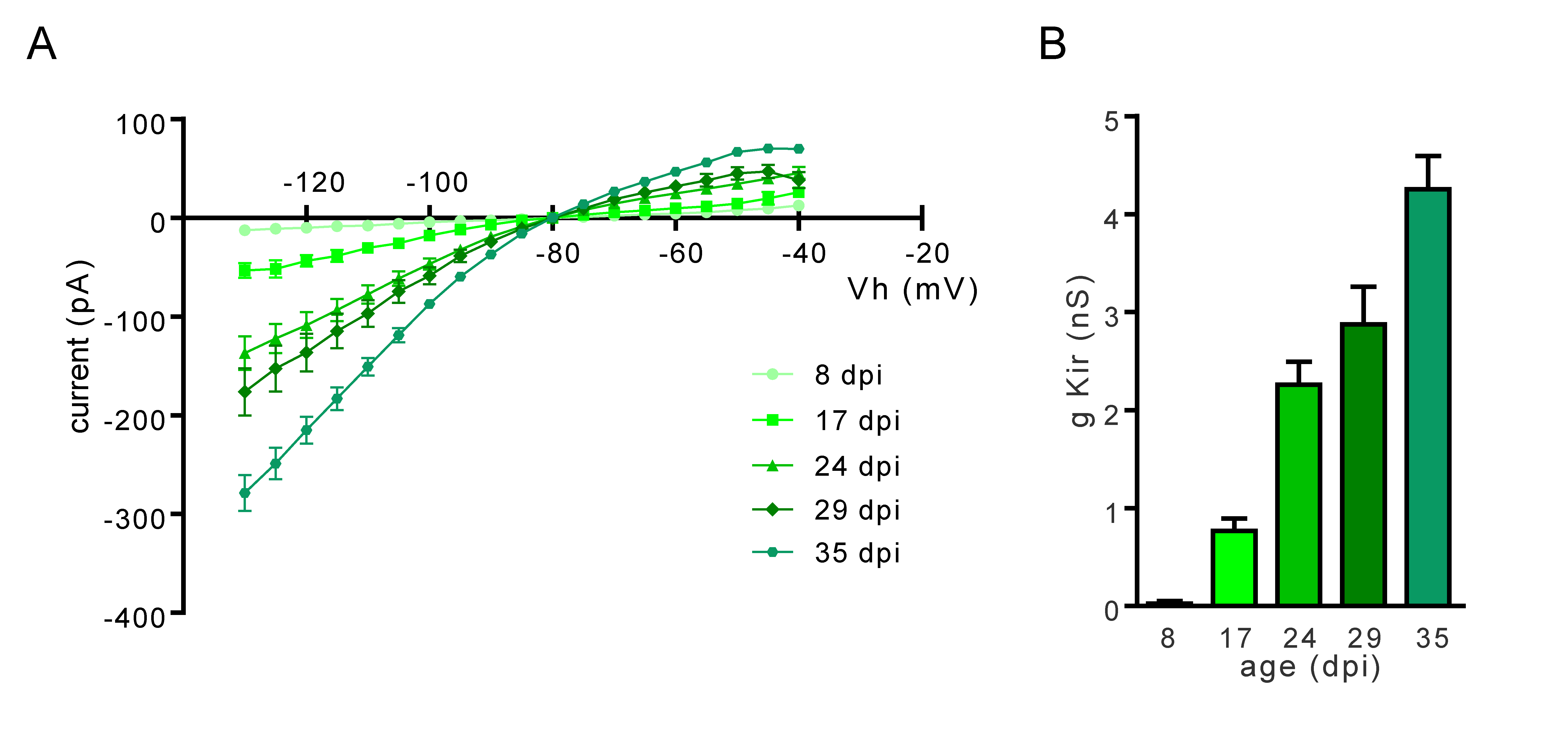

Supplement: Figure S3 — Ontogeny of Kir currents in adult-born DGCs. (A) Average I–V curves, with N = 13 (8 dpi), N = 14 (17 dpi), N = 18 (24 dpi), N = 12 (29 dpi) and N = 15 (35 dpi). (B) Kir conductance calculated for the experiments shown in (A). (1.10 MB TIF) [file pone.0005320.s003.tif]

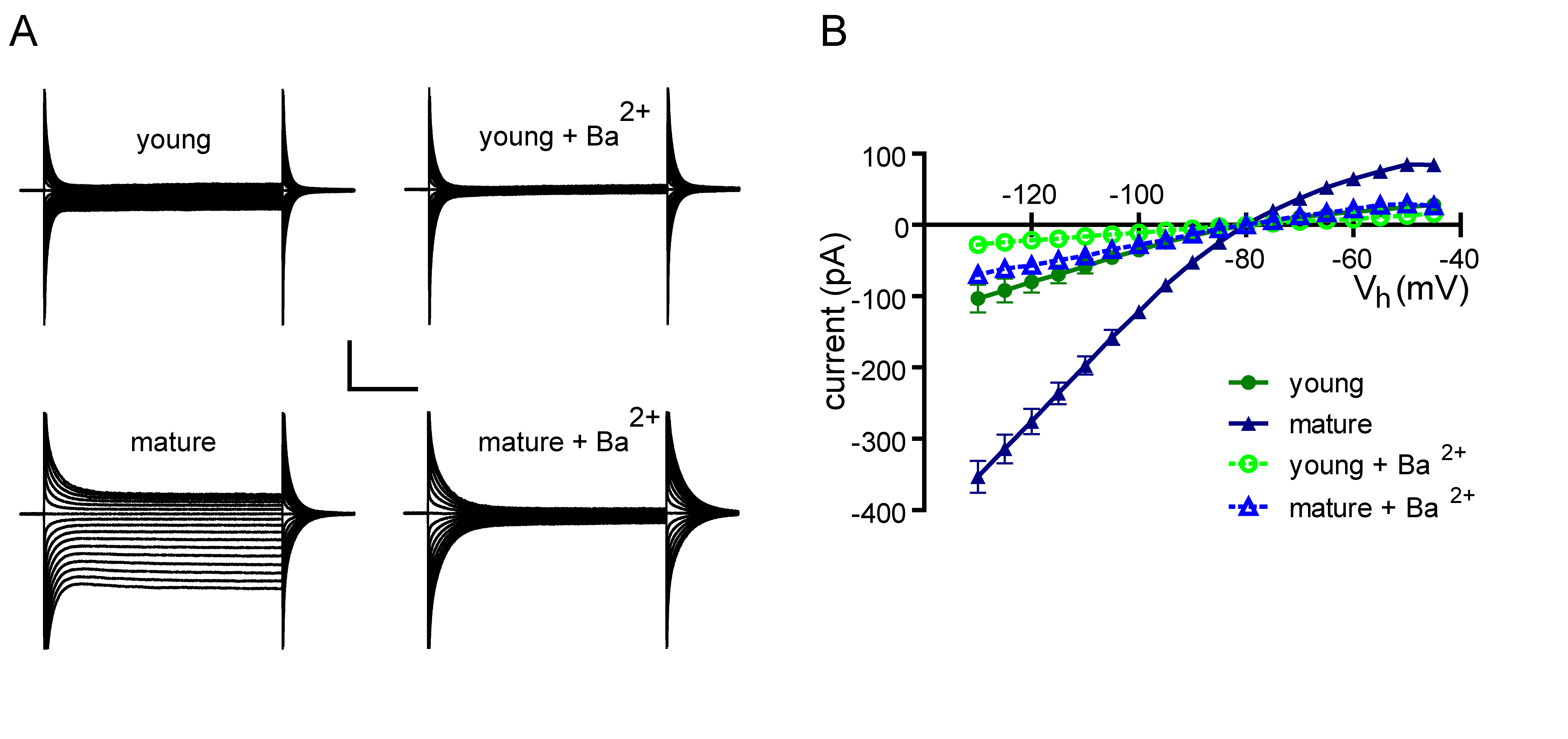

Supplement: Figure S4 — Kir blockade by extracellular Ba2+. (A) Example current traces of young (25 dpi) and mature neurons recorded in the absence (left) or presence (right) of BaCl2 (200 µM). Voltage steps from −45 to −130 mV (step 5 mV, 100 ms) for a 25 dpi and a mature neuron. Scale bars: 200 pA, 30 ms. (B) Mean I–V plots obtained from N = 24 (young), 8 (young+Ba2+), 28 (mature) and 23 (mature+Ba2+). (0.96 MB TIF) [file pone.0005320.s004.tif]
